# Beliefs underlying Women’s intentions to consume alcohol

**DOI:** 10.1186/s12905-016-0317-3

**Published:** 2016-07-13

**Authors:** Helen M. Haydon, Patricia L. Obst, Ioni Lewis

**Affiliations:** Centre for Accident Research and Road Safety – Queensland, K Block, Queensland University of Technology, Kelvin Grove, Queensland 4059 Australia; School of Psychology and Counselling; O Block, in Kelvin Grove, Queensland University of Technology, Brisbane, Queensland 4059 Australia; Centre for Accident Research and Road Safety – Queensland, K Block, Queensland University of Technology, Kelvin Grove, Queensland 4059 Australia

**Keywords:** Women’s health, Alcohol, Theory of Planned Behavior, Critical beliefs, Drinking behavior, Women’s drinking

## Abstract

**Background:**

Changing trends demonstrate that women, in a number of economically-developed countries, are drinking at higher levels than ever before. Exploring key targets for intervention, this study examined the extent to which underlying beliefs in relation to alcohol consumption predicted intentions to drink in three different ways (i.e. low risk drinking, frequent drinking and binge drinking).

**Methods:**

Utilizing a prospective design survey, women (*N* = 1069), aged 18–87 years, completed a questionnaire measuring their beliefs and intentions regarding alcohol consumption. Then, two weeks later, 845 of the original sample, completed a follow-up questionnaire reporting their engagement in the drinking behaviors. A mixed design ANOVA was conducted to examine potential differences between women of different age groups (18–24, 25–34, 35–44, 45–54, 55 years and above) and their intentions to engage in the three different drinking behaviors. Based upon The Theory of Planned Behavior, critical beliefs analyses were carried out to identify key determinants underlying intentions to engage in the three different drinking behaviors.

**Results:**

Significant effects of age were found in relation to frequent and binge drinking. The critical beliefs analyses revealed that a number of behavioral, control and normative beliefs were significant predictors of intentions. These beliefs varied according to age group and drinking behavior.

**Conclusions:**

Previously unidentified key factors that influence women’s decisions to drink in certain ways have been established. Overall, future interventions and public policy may be better tailored so as to address specific age groups and drinking behaviors.

**Electronic supplementary material:**

The online version of this article (doi:10.1186/s12905-016-0317-3) contains supplementary material, which is available to authorized users.

## Background

Alcohol misuse continues to pose widespread public health problems [1, 2]. Changing trends indicate that women are drinking at higher levels than ever before [3, 4]. Research demonstrates gender differences in alcohol consumption and alcohol-related harms wherein women, compared to men, are more at risk for detrimental physical, medical, social and psychological effects of at-risk consumption [5]. Amount, frequency and duration of alcohol consumed, should be considered when measuring the effects and risks of alcohol consumption [6, 7]. For instance, the positive linear association between women’s drinking with increased risk of breast cancer emphasizes the need to examine regularity of alcohol intake [8, 9].

The trend of women’s increased alcohol consumption is now evidenced across all ages, yet most research has focused on younger women [5, 10, 11] to the exclusion of older women. Further, the underlying decision-making that leads to women’s choices regarding such consumption requires further examination. This gap is critical given that recent data has suggested that risky drinking in this older demographic is becoming an emerging issue in Australia [3, 12–15].

**Fig. 1:**
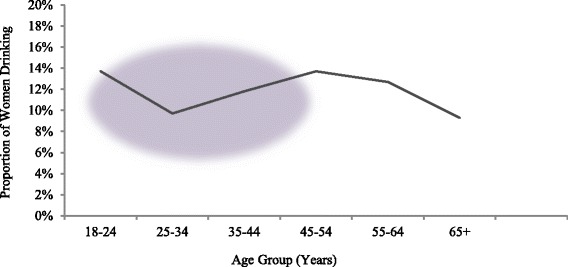
Proportion of women in Australia drinking at risky and high risk levels (ABS, 2009, 2012)

Australian data shows an anomaly in the proportion of women drinking at risky or high risk levels between the ages of 18 years and 55 years where an apparent dip in the proportion of women drinking at risky or high risk levels between the ages of 25 to 34 years (assumed to be associated with women being of prime child-bearing age) is observed [14] (See Fig. 1). The proportion of women drinking at risky and high risk levels was up as far as 14 % for women in the age groups 18–24 years and 45–54 years [3]. Additionally, the proportion of women aged 40 to 69 years who were drinking at long term risk levels had increased between 2010 and 2013 [15]. Research to facilitate understanding of this trajectory and examine potential differences between the age groups in underlying motivations to drink is needed.

Differing patterns of alcohol use according to age and gender [4, 16], along with the associated risks, substantiate the need to further understand the psychological determinants that relate to drinking behaviors across women of all ages. Hence, examination and understanding of underlying factors associated with women’s decision making around alcohol consumption could help to better focus prevention and intervention measures to reduce alcohol-related harms.

### Critical beliefs in the theory of planned behavior

The Theory of Planned Behavior (TPB; [17, 18]) is a useful model to gain understanding of women’s decision-making with regard to their drinking behaviors. The TPB is acknowledged as a stable theory which can predict a wide variety of health and social behaviors including alcohol consumption [19–22]. The TPB’s premises are threefold. Firstly, intentions to perform a particular behavior and perceived behavioral control (PBC) are direct predictors of the behavior. Secondly, intentions are directly determined by attitude (an individual’s positive and negative evaluations of the behavior), subjective norm (an individual’s perceptions of the social pressure to engage in the behavior), and PBC (perceptions of one’s ability to undertake the behavior). Thirdly, these three constructs (attitude, subjective norm and PBC) are underpinned by a set of beliefs (herein termed as critical beliefs) wherein: attitude is influenced by behavioral beliefs (costs or benefits of enacting the behavior); subjective norm is informed by normative beliefs (important others’ approval or disapproval with regard to the behavior); and PBC is a function of control beliefs (barriers or facilitators to engaging in the behavior).

Research has shown that these critical beliefs underlying attitude, subjective norm and PBC may help inform the content of interventions to reduce harmful behaviors [20, 23]. Currently, however, such beliefs appear to have been somewhat overlooked in the alcohol and TPB research [20]. Studies have examined the relationship of critical beliefs that underlie various health behaviors to propose targeted health behavior interventions [24, 25]. Such interventions could reduce alcohol-related related risks [20, 24].

### Aims

Drawing on the TPB as a useful framework for informing the development of interventions, the current study had two principle objectives. The first objective was to elicit women’s beliefs regarding alcohol consumption in general. Second, the study aimed to examine the strength of the relationships between these critical beliefs and intentions to drink in three different ways (i.e. low risk drinking, frequent drinking and binge drinking).

## Methods

### Participants

Participant recruitment was initiated by a media release (e.g. nationwide radio and newspaper interviews) and through subsequent snowballing. Additionally, there was recruitment of first year students at a large Australian university. Flyers requested participation of all women living in Australia regardless of whether they drank alcohol or not. Dissemination of online questionnaires (*N* = 1049) was via social networking sites (e.g. Twitter™, Facebook™), email lists and websites. Hard copy questionnaires (*N* = 20) were provided to women who requested them.^1^ The study’s three inclusion criteria were being female; aged 18 and over; and living in Australia.

Women (*N* = 1069) residing in Australia and aged between 18 and 87 years (*M* = 35, *SD* =. 13.7) completed the questionnaire. Of these 1069 women, 845 (79 %) also completed the follow up questionnaire assessing their self-reported engagement in low risk, frequent and binge drinking. To examine potential differences across age groups in the underlying beliefs regarding drinking, the responses were grouped according to the following ages: 18 to 24 years (30.6 %); 25 to 34 years (25.8 %); 35 to 44 years (20.5 %); 45 to 54 years 13.8 %); and 55 years and above (9.3 %). This age group categorization is consistent with the fluctuating drinking trends [3, 14]. In terms of education; 28 % of the sample had achieved high school level, 16.1 % trade certificate, 31.5 % undergraduate degree, and 24.4 % postgraduate degree. Of the participants; 50.4 % were married/ defacto, 10.2 % were partnered but not living together, 32.1 % had never been married, and 7.3 % were divorced/ widowed.

### Design and procedure

The critical beliefs examined in the current study were based on responses generated from a beliefs elicitation study wherein semi-structured interviews with thirty-five women (aged 18 to 55 years) explored beliefs underpinning drinking in general [26]. The current study utilized these identified general beliefs about alcohol consumption to explore the association between these beliefs and intentions to engage in low risk drinking, frequent drinking, or binge drinking. The prospective design survey also allowed assessment of which beliefs were direct determinants of actual self-reported drinking behaviors. Hence, as a final step, all of the significant beliefs (from Phase 1 which predicted intentions) were also regressed on actual drinking behaviors (Phase 2). Ethical approval was obtained from the university’s Human Research Ethics Committee. All participants were provided with an information sheet. Questionnaire submission was deemed as consent.

### Measures

#### Main questionnaire: assessing intentions to drink (Phase 1)

Questions assessing intentions to drink, and the associated underlying critical beliefs, were based on the standard TPB self-report format [27–29]. All items were scored on 7-point Likert scales. The intentions scales ranged from “1–strongly disagree” to “7–strongly agree” and pertained to each of the targeted behaviors. The beliefs scales ranged from “1–extremely unlikely” to “7–extremely likely” and pertained to “Thinking about your attitudes and beliefs about drinking alcohol in general.” Sociodemographic information was also collected. Please see the attached file displaying the Additional file 1.

#### Drinking behaviors

Participants were provided with a standard drinks guide and definitions of the three target drinking behaviors as follows: Low Risk—drinking no more than one or two drinks occasionally; Frequent Drinking—drinking 6 or more days in the one week; Binge drinking—drinking 5 or more standard alcoholic drinks on any one occasion.

#### Intention

For each of the three behaviors, five items measured intention (e.g. “Do you agree that in the next 2 weeks… it is likely that I will engage in drinking on 6 or more days in a typical week?”).

#### Normative beliefs

Five items measured normative beliefs, with normative referents including one’s partner, family, friends and professional/work colleagues. The fifth item stated, “Having concerned family members or friends would stop me from drinking.”

#### Behavioral beliefs

Asking participants, “How likely is it that the following would occur as a result of you drinking alcohol?” 16 items (8 benefits and 8 costs) measured behavioral beliefs. (e.g. “Having a drink containing alcohol would… ‘Help me to talk with others’ [benefit] or ‘Make me sick in the short term’ [cost].”).

#### Control beliefs

Providing the context, “How likely is it that the following would STOP you from drinking alcohol?” participants were asked 12 items to assess barriers to drinking (e.g. “The following factors would stop me from drinking …Being pregnant or breastfeeding.”). With the precursor “How likely is it that the following would INCREASE the possibility of you drinking alcohol?” 13 items assessed facilitators of drinking (e.g. “The following would increase the chances of me drinking…If it was my birthday.”).

##### Follow-up questionnaire: assessing engagement in behaviors (phase 2)

Two weeks after Phase 1 was conducted participants were asked to report their drinking behavior over the interim period from the Phase 1 survey. Participants were asked to report the quantity of alcohol they had consumed, “In the last 2 weeks if you drank alcohol, how many standard alcoholic drinks did you have on a typical drinking occasion?” which assessed the amount that they drank and was compared with intentions to low risk drink. Frequency of drinking was assessed by asking participants “In the last 2 weeks, on how many days did you have a drink containing alcohol?” Finally, consistent with the main survey’s assessment of binge drinking, participants were asked to select “Never, Once, Twice, 3 times, 4 times or 5 or more times in response to the question, “In the last 2 weeks how often did you have 5 or more standards drinks on any one occasion?” This allowed an examination of the extent to which the women were engaging in binge drinking. Please see the attached file displaying the Additional file 2.

### Analysis

A three step critical beliefs analysis was undertaken as guided by von Haeften, Fishbein, Kasprzyk and Montano [30]. Firstly, the Pearson correlation matrices were examined to identify beliefs that were significantly correlated with the intentions to low risk drink, frequent drink and binge drink (see Additional file 3). Secondly, the significant beliefs from the Pearson correlation matrices were entered according to belief type (i.e. behavioral, normative and control) into stepwise regressions. These regressions allowed identification of the critical beliefs that independently contributed to intentions to drink (low risk, frequent and binge). Finally, all of the significant beliefs from the stepwise analyses were put into three (one for each behavior) final multiple regressions to assess the significant contributors to intentions. A final analysis then assessed which of the beliefs that had emerged as significant predictors of intentions were also associated with actual drinking behavior.

## Results

### Comparing age groups within intentions to low risk drink, frequent drink and binge drink—the ANOVA analyses

A 5 x 3 mixed design ANOVA was conducted to examine the differences between the age groups’ engagement in the three different drinking behaviors of low risk, frequent drinking and binge drinking. Age was operationalised by dividing participants into the age groups: 18 to 24 years (*n* = 324); 25 to 34 years (*n* = 278); 35 to 44 years (*n* = 217); 45 to 54 years (*n* = 150); and 55 years and above (*n* = 100). Multivariate results revealed a significant interaction between age and intention to drink, Wilks’ E = .80, *F*(8, 2100) = 30.57, *p* < .001, partial ŋ^2^ = .10.

The univariate analyses of the simple effects of age on each of the drinking behaviors (See Table 1) showed no significant effect of age on intentions to low risk drink, but significant effects of age on intentions to frequent and binge drink. Examination of the post hoc pairwise tests with an adjusted alpha level of .025, to control for familywise Type 1 error, revealed that older women (45–54 and 55 years and above), were more likely to intend to frequent drink compared with all younger counterparts. Comparing between age groups on binge drinking, 18 to 24 year olds were more likely to intend to binge drink compared with all of the other age groups. Each age group progressively, from youngest to oldest, was less likely to binge drink as they got older, with the exception of women aged 35 to 44 years and 45 to 54 years where there was no significant difference. These analyses indicate the particular risky behaviors that were most relevant to each age group.

**Table 1:** Comparison between age groups on intentions to low risk, frequent and binge drink

Intentions to drink	18 to 24 years *M* (*SD*)	25 to 34 years *M* (*SD*)	35 to 44 years *M *(*SD*)	45 to 54 years *M* (*SD*)	55 years and above *M *(*SD*)	*F*	Sig.	partial ŋ^2^
Low risk	4.83 (1.51)	4.93 (1.41)	4.62 (1.56)	4.69 (1.51)	4.87 (1.44)	1.61	.169	.006
Frequent	1.73 (0.95)	1.81 (1.06)	2.10 (1.20)	2.54 (1.56)	2.40 (1.40)	16.86	<.001	.060
Binge	3.95 (1.16)	3.65 (1.14)	3.29 (1.15)	3.14 (0.99)	2.70 (0.82)	33.10	<.001	.112

### Critical beliefs underpinning intentions—the regression analyses (phase 1)

Additional file 3 shows the significant bivariate correlations between the critical beliefs and intentions to low risk drink (Additional file 3: Table A1), frequent drink (Additional file 3: Table A2) and binge drink (Additional file 3: Table A3). The results of the stepwise regression analyses on the significant beliefs are shown in Appendix B, which displays the significant predictors of intention to low risk drink (Additional file 3: Table B1), frequent drink (Additional file 3: Table B2) and binge drink (Additional file 3: Table B3) for each age group. The results from the final multiple regression analyses which revealed the significant critical beliefs predicting intentions are presented and discussed below.

#### Low risk drinking

The critical beliefs that significantly predicted intentions to low risk drink across age groups are presented in Table 2.

**Table 2:** Women’s critical beliefs that significantly predict intentions to low risk drink and amount drunk across age groups

		*Intentions*			*Behavior*		
Age	Women’s beliefs	B	β	*sig*	B	β	*sig*
18–24	Control Beliefs						
	Values (e.g. religious, health, sport)	–.08	–.10	.038	–.18	–.11	.065
	If I am on holidays	.34	.33	<.001	.08	.04	.622
	If I am at a nice restaurant	.13	.14	.005	.00	.00	.975
	If it is my birthday	.25	.24	<.001	.69	.34	<.001
25–34	Behavioral Beliefs						
	Interferes with my family life	–.20	.23	<.001	.06	.05	.511
	Control Beliefs						
	If I am at a nice restaurant	.22	.24	<.001	.03	.02	.756
	If it is my birthday	.19	.20	.002	.29	.20	.009
35–44	Behavioral Beliefs						
	Make me feel relaxed	.17	.15	.038	.08	.06	.462
	Interfere with my family life	–.23	–.26	<.001	.07	.07	.321
	Control Beliefs						
	Stressful week	.13	.16	.027	.22	.26	.003
	If I am on holidays	.24	.23	.003	.33	.29	.001
45–54	Behavioral Beliefs						
	Make me feel relaxed	.31	.28	.001	.45	.38	<.001
	Make me sick in the short term	–.16	–.21	.004	.10	.11	.218
	Control Beliefs						
	Not able to control my behavior	.29	.28	<.001	.07	.06	.542
	Being a mother	–.16	–.18	.014	–.17	–.17	.081
	If it is Christmas or New Year (Cultural celebration)	.18	.19	.019	–.05	–.04	.672
55 and above	Behavioral Beliefs						
	Interfere with my family life	–.16	–.21	.014	–.09	–.09	.399
	Normative Belief						
	Spouse/ partner	.12	.24	.005	–.05	–.08	.464
	Control Beliefs						
	If I am on holidays	.23	.30	<.001	.38	.43	.001
	Access to wineries/ wine clubs	.25	.34	.005	–.17	–.20	.090

#### Frequent drinking

The critical beliefs that significantly predicted intentions to frequent drink across age groups are presented in Table 3.

**Table 3:** Critical Beliefs that significantly predict intentions to frequent drink and frequency of drinking across age groups

		*Intentions*			*Behaviors*		
Age	Women’s Beliefs	B	β	*sig*	B	β	*sig*
18–24	Behavioral Beliefs						
	Cost more than can afford	.06	.11	.048	–.03	–.03	.633
	Control Beliefs						
	Having to drive	–.27	–.19	.001	–.53	–.14	.019
	Stressful week	.06	.13	.040	.44	.38	<.001
25–34	Behavioral Beliefs						
	Help me to unwind	.16	.20	.001	.85	.34	<.001
	Normative Beliefs						
	Concerned family or friends	–.15	–.20	.002	–.57	–.24	<.001
	Control Beliefs						
	Pregnant or breastfeeding	–.26	–.18	.002	–.57	–.11	.092
35–44	Control Beliefs						
	Pregnant or breastfeeding	–.27	–.22	.001	–.64	–.13	.095
45–54	Behavioral Beliefs						
	Make me feel relaxed	.21	.18	.046	1.18	.38	<.001
	Control Beliefs						
	Having health issues	–.40	–.26	.001	–.87	–.20	.019
55 and above	Normative Beliefs						
	Friends	.13	.19	.046	.62	.25	.013
	Control Beliefs						
	Family commitments I must keep	–.24	–.27	.006	.01	.00	.98
	Stressful week	.14	.20	.047	1.02	.44	<.001

#### Binge drinking

The critical beliefs that significantly predicted intentions to binge drink across age groups are presented in Table 4.

**Table 4:** Women’s critical beliefs significantly predicting intentions to binge drink and engagement in binge drinking for each age group

		*Intentions*			*Behavior*		
Age	TPB Construct	B	β	*sig*	B	β	*sig*
18–24	Control Beliefs						
	Being uncomfortable (e.g. feelings in an environment)	–.10	–.14	.002	–.04	–.06	.375
	Short term side effects of alcohol (hangovers)	–.17	–.27	<.001	–.09	–.16	.018
	If tolerance was better (e.g. less “suffering” the next day)	.11	.18	.001	.02	.04	.600
	If I didn’t have children	.09	.14	.008	.07	.12	.117
	If it was my birthday	.16	.19	.001	.09	.13	.092
	If I was close to wineries or wine clubs	–.06	–.10	.026	.05	.10	.148
25–34	Behavioral Beliefs						
	Help me to have fun	.11	.14	.015	.14	.17	.010
	Control Beliefs						
	Having non-family commitments I must keep	–.15	–.15	.010	–.19	–.16	.011
	Short term side effects of alcohol (hangovers)	–.16	–.24	<.001	–.21	–.29	<.001
35–44	Control Beliefs						
	Having health issues	–.21	–.13	.045	–.27	–.18	.015
	Short term side effects of alcohol (hangovers)	–.13	–.17	.008	–.14	–.19	.011
	If I am on holidays	.15	.20	.033	.12	.17	.053
	If there is an important sporting event	.17	.33	<.001	.10	.20	.023
	If I was close to wineries or wine clubs	–.09	–.16	.034	–.05	–.10	.257
45–54	Behavioral Beliefs						
	Make me feel relaxed	.16	.23	.007	.03	.05	.599
	Control Beliefs						
	Short term side effects of alcohol (hangovers)	–.12	–.18	.035	–.12	–.18	.060
	Values (e.g. religious) not aligned with drinking	–.13	–.25	.001	–.10	–.18	.064
	If drinking venues were in walking distance	.08	.16	.035	.14	.26	.003
55 and above	Behavioral Beliefs				*Only 10 women in this age cohort reported engagement in binge drinking. Hence a regression for this age group and behavior could not be conducted.*		
	Make me feel irresponsible	.10	.20	.046			
	Control Beliefs						
	Short term side effects of alcohol (hangovers)	–.15	–.25	.008			

### Critical beliefs underpinning engagement in drinking behaviors (Phase 2)

Overall, the majority of beliefs which were found to be significantly predictive of intentions to drink alcohol, were often also significantly associated with actual drinking behavior. See Tables 2, 3 and 4 for the results of the regression analyses.

## Discussion

The current study examined the extent to which women’s alcohol-related behavioral, normative, and control beliefs predicted intentions, and subsequent engagement in low risk drinking, frequent drinking and binge drinking across age cohorts. It was evident that women’s intentions to drink differed as a function of their age. Specifically, younger women were more likely to report intentions to binge drink than older women and older women were more likely than younger women to report an intention to drink frequently. Although the findings are consistent with women’s drinking patterns found in Australia [31], they do highlight the need to further investigate the risks associated with frequent drinking particularly for older women. Frequent drinking has its own set of risks as it can: result in higher alcohol tolerance levels which can lead to increased volumes to gain the desired effect (e.g. relaxation); be related to increased likelihood of frequent binge drinking [32]; and even in the absence of binge drinking can still represent high volumes of alcohol intake across a lifetime and long term harms [33]. Further, if typical quantities of alcohol consumed are often underreported [34] and alcohol is frequently present in women’s lives, such habituated drinking brings with it greater exposure to long term harms.

Overall, a range of behavioral beliefs, control beliefs, and normative beliefs were found to predict women’s intentions to drink, and the majority of these were also shown to be predictive of actual alcohol consumption. Consistent with previous studies, identification of such beliefs is significant to the extent that they may guide the development of targeted interventions, such as public education and health promotion messages [20, 23]. It follows then, that identifying the key beliefs underpinning women’s intentions, and actual behaviors, to engage in drinking could inform message development. Further specific examples will be provided in this discussion but, just as one overall example, identifying the perceived disadvantages associated with engaging in binge or frequent drinking would provide important insights into perceived negative aspects of drinking that messages could emphasise to ultimately reduce alcohol related risks.

Within the final regression results, only three normative beliefs were found to significantly predict women’s intentions to drink (i.e., low risk, Table 2 and frequent, Table 3), and two of these were also predictive of actual behavior. Normative beliefs were found to predict intentions to frequent drink for women aged 25 to 34 years. Specifically, the findings revealed that perceptions of having family or friends concerned about one’s drinking reduced one’s intentions to frequent drink (Table 3). Notably, for women aged 55 and over, perceptions of their friends’ alcohol-related expectations were positively associated with intentions to frequent drink (Table 3). For this age cohort, normative beliefs regarding spouses’ alcohol-related views also significantly predicted women’s intentions to low risk drink (Table 2). These findings highlight age and possible generational differences in regards to the importance of different normative referents around alcohol. Such insight highlights how, for instance, age-based educational messages could be designed to either emphasize those most relevant normative influences who disapprove of one’s drinking or to challenge those normative referents perceived as positively influencing one’s drinking. The current findings build upon previous evidence of partner influence as a risk factor for alcohol-related harms [35] and the efficacy of normative feedback interventions [36], all of which highlight the importance in gaining an understanding of such potential intervention targets.

Until now, the beliefs underlying intentions to engage in the specific drinking behaviors have not been widely examined. In the discussion below, beliefs specific to age groups are highlighted and then commonalities across age groups are discussed. These results greatly assist in identifying specific age appropriate targets for intervention and more general points of intervention applicable to women of varying ages.

### Low risk drinking

There were no age-related differences of women’s intentions to drink on only one or two occasions. Women’s intentions to low risk drink were influenced by beliefs that varied somewhat according to age group, but there were some patterns evident. The results indicated that for women in all age groups from 25 and above, family concerns significantly affected their intentions to engage in low risk drinking. On inspection of the behavioral beliefs influencing intentions reported by women aged between 25 and 44, the age cohort most likely associated with raising a family, it was revealed that even having an occasional drink is perceived as interfering with family life and therefore this aspect was a perceived disadvantage. For women aged 45 to 54, being a mother was perceived by participants in this study as a significant barrier to their intentions to low risk drinking (i.e. control belief - barrier).

Perceived benefits and facilitators of consuming alcohol were identified as significant predictors of intentions for women aged between 35 and 54. In particular, these beliefs included perceiving an advantage of drinking as being it helping one to relax (behavioral belief) and a perceived facilitator of drinking being it reduces stress (control belief), and were also significantly predictive of women’s reported drinking behavior. These findings may be reflective of a time of life where family and work commitments are high and a need to for work, life and family balance is needed [37, 38]. Although these results refer to intentions to low risk drink and thus are indicative of a safer way to drink, there may be value in challenging perceptions that alcohol is an appropriate and acceptable stress management technique. Across all age groups, one of the most consistent motivations which predicted having an occasional drink related to special occasions and celebrations (e.g. birthdays, Christmas, holidays). It seems that the presence of special occasions and celebrations is a strong and consistent facilitator of women’s intentions to low risk drink and engagement in such drinking behavior, irrespective of one’s age. Interestingly, family concerns did not emerge as significant predictors of actual drinking, indicating that these beliefs may be more related to planning and intentions than behavior itself, while beliefs around drinking being a source of stress relief and celebration were associated strongly with both intention and behavior.

### Frequent drinking

Older women, aged 45 years and above, were more likely to report an intention to drink frequently compared with younger women, a finding that is consistent with other research [16, 31]. Examination of motivating beliefs underlying these women’s intentions to drink on six or more days per week, revealed beliefs relating to: relaxation (behavioral belief—benefit) and stress relief (control belief—facilitator); family influence (normative) and responsibility (control belief—barrier); and health issues (control belief—barrier) (Table 3). For these older women, behavioral beliefs in terms of perceived advantages of drinking that “made them relax” (aged 45 to 54) and control beliefs with regard to facilitating their drinking when they are “having a stressful week” (aged 55 and above) were associated not only with intentions to frequent drink, but also were strong predictors of actual drinking frequency. In regards to normative beliefs, women aged 55 years and above also reported that they expected friends to be supportive of frequent drinking. The only two barriers to frequent drinking which were identified related to health issues (aged 45 to 54) and family commitments (55 and above). Health and family were a concern for other age groups too.

Being pregnant or breastfeeding was reported by women aged between 25 and 44 as a significant control belief associated with reduced reported intentions to frequent drink, but this belief was not a significant predictor of actual drinking frequency. These findings are consistent with data showing that women are having children at an older age, compared with previous generations [37, 38], and that parenthood affects the frequency of women’s drinking more so than men’s drinking [39].

With the exception of the 35 to 44 years age cohort, all of the age groups reported a perceived advantage of alcohol as it offering a means to relax (behavioral beliefs—benefit) and to be used as a stress management tool (control beliefs—facilitator), both of these beliefs were also significant predictors of frequent drinking behavior. Such consistent findings across age cohorts indicate a need to address stressors specific to women and promote alternative healthy stress management techniques. Although Australian drinking guidelines do not explicitly warn against drinking on a daily basis they indicate long term health risks associated with high frequency of drinking [7]. Further, for women, even moderate drinking levels are associated with certain health risks such as breasts cancer [40, 41] and drinking patterns and associated beliefs can be predictive of later drinking behaviors that may be risky [42, 43]. The current findings support the idea that women are considering alcohol as an advantage in terms of its use as a relaxation and stress management tool. Hence, promotion of alternative stress management tools and awareness regarding long term risk exposure needs to be considered in future public health policy. As with the findings for low risk, this family-oriented belief was not significantly predictive of behavior, again highlighting the subtle effect of these beliefs on planning and intention, while stress reduction beliefs were predictive of both intention and behavior.

### Binge drinking

In line with drinking patterns in many developed nations [1, 3, 31, 44], results from this study indicated that younger women were more likely to report intentions to binge drink compared with older women (Table 4). When investigating the beliefs predicting such intentions, the results showed that different underlying beliefs were predictive of intentions to binge drinking compared to the other behaviors, and for the most part, were also specific to different age groups. In particular, the findings highlighted the important influence of a broad range of behavioral and control beliefs.

For the women aged 18 to 24 who were most likely to binge drink, a number of control beliefs were found to be significant predictors, specifically beliefs which would facilitate their intentions to drink more than four drinks on any one occasion. These facilitators related to: birthday celebrations, if their alcohol tolerance was better, accessibility to wineries and wine clubs; and if they did not have children. Possibly, this latter facilitator is tapping into younger mothers who are no longer able to binge drink because of their responsibility as a mother, but further investigation is needed to clarify this result as this suggestion is only speculative. With increasing age, an interesting finding was the finding that the behavioral belief of “help me to have fun” was a significant predictor of intentions to binge drink in the 25 to 34 year olds but, not for 18 to 24 year olds. Previous research has identified elements of fun and excitement as motivating factors for young women (aged 18 to 30 years) [45] and young people (aged 18 to 24 years) [46] in their decisions to binge drink. The need to address young women’s perceptions of binge drinking as fun, whilst negating the associated risks, is underlined.

For women aged 35 to 44, a significant control belief which would prevent binge drinking which emerged related to health issues. In line with women’s drinking patterns [3, 31], this finding may indicate the transition from binge drinking to frequent drinking as women’s health becomes less tolerant of binge drinking over time. The transition away from binge drinking is evident with the 55 years and older group. This cohort reported no significant benefits or facilitators predicting intentions to binge drink. Instead, perceived barriers such as short term effects of alcohol and behavioral evaluations that binge drinking “makes me feel irresponsible” were found to be significant predictors of intentions to binge drink. Such results highlight why this cohort is less likely to binge drink and further implies the existence of differing social norms with regard to women and the acceptability of binge drinking.

The main consistency in regards to the significant predictors of binge drinking across all age groups was that of the control belief of short term side effects (e.g. hangovers) which was associated with significantly lower intentions to binge drink. Avoiding short term side effects was also significantly associated with self-reported engagement in binge drinking behaviors for women aged 18 to 44 years old. It would seem prudent then to target interventions to build upon women’s perceptions about these beliefs, especially for the younger women who tend to binge drink at higher rates. An anomaly in the results emerged for the women aged 18 to 24 years and 35 to 44 years with regard to accessibility of “wineries or wine clubs”. It was expected that the control belief “If I was close to wineries or wine clubs” would be a factor that facilitated intentions to binge drink, not the negative relationship that was actually found. One reason for this may be that context is the important factor wherein wineries are not perceived as venues in which to binge drink. Drinking context has been found to be an influential factor on drinking behaviors [1, 47, 48].

### Implications

#### Across age groups

Across behaviors and age groups it became evident that two beliefs consistently underpinned probable targets of intervention. Firstly, although beliefs varied across age groups and drinking behaviors, beliefs surrounding alcohol as a tonic to a stressful week and a way to unwind and relax, were consistent across age groups and across behaviors. Implications from this current study call upon a focus on women’s decision-making regarding alcohol and stress-relief. The findings highlight the role that alcohol and drinking has taken on in the lives of contemporary women as a means of relieving stress. Furthermore, the findings suggest that there may be value in both challenging the notion that alcohol is an important and regular means by which to reduce stress and instead highlight healthy alternatives to stress relief including, for instance, going for a walk or taking a bath. Secondly, the perceived short term effects of binge drinking was found to be a barrier to engaging in this behavior across all age groups. Alongside this finding, however, the finding that the behavioral belief of “cause ill-health in the long term” was not a significant predictor in any of the regression models is, arguably, most noteworthy. It is noteworthy given that it suggests that many women are not perceiving and/or are have limited awareness of the long term health risks associated with alcohol consumption, highlighting the need to raise awareness in regard to this issue [21].

#### Frequent drinking

For the older women intending to drink more than five days in a week, behavioral and control beliefs regarding relaxation and stress relief, respectively, and perceived peer expectations (normative beliefs) may be of value in informing intervention content. The belief that friends approve or expect a woman to drink frequently needs further investigation. Previous examinations have found misperceptions regarding normative beliefs around alcohol consumption, whereby individuals may well overestimate the perceived peer expectations to drink [48]. Consequently, this misperception represents an important normative belief which needs to be challenged in targeted messages.

Although the current study did not investigate drinking locations, other studies have raised concerns about older women’s alcohol related risk because of the way in which they drink [5]. If older women have an increased likelihood of frequent drinking in the privacy of their homes there may be less opportunity for detection of drinking problems [5]. As such, older women are thus exposed to a different set of risks compared to their younger counterparts, who may be more visible in public spaces where acute alcohol risk is more pertinent [5]. Hence, reiterating the need for a different set of interventions and public health messages for older women compared with younger women.

#### Binge drinking

In conjunction with other TPB research outlining the importance of beliefs in devising and informing targeted interventions [20, 24], this current study’s results suggest, that for the younger women who engage in binge drinking, it will likely be of value to devise messages that target the beliefs that facilitate binge drinking. Public health promotions depicting young women as having fun and celebrating birthdays without binge drinking could raise awareness and combat the belief that heavy drinking is the main way to celebrate. Research has identified cultural and normative influences on young people to celebrate birthdays, particularly milestone birthdays (e.g. 18th and 21^st^), with excessive alcohol consumption [49–51]. Such notions could be challenged with increased public awareness and public health campaigns depicting healthier fun alternatives.

As discussed above, emphasizing the short term health risks and side effects of drinking more than four drinks on any one occasion could help to consolidate such a barrier to binge drinking for women of all ages. In line with this suggestion, young women reported that if their tolerance for alcohol meant that they would suffer less adverse reactions to binge drinking, it would facilitate more binge drinking. This belief needs to be challenged, but also outlines the presence of a desire to binge drink. It also raises the point that if women had a high tolerance level then potentially their consumption would increase. This was a theme that arose in the beliefs elicitation study [26]. Hence, women’s beliefs around tolerance of alcohol and ways to negate short term risk needs further investigation.

### Strengths and limitations

This current study has a number of strengths including: the examination of women’s drinking across a broad age range and across different drinking behaviors; having a large sample of women; and using a well-established theoretical framework to identify critical beliefs underlying intentions to engage in drinking behaviors. Furthermore, this study examined differences in beliefs across age and behavior that can inform targeted interventions. One limitation is the cross-sectional nature of the study and hence results must be interpreted as such. Furthermore, whilst possible intervention foci have been identified, further research is needed to assess the efficacy of interventions targeting beliefs to reduce alcohol-related risks.

Although, the TPB is a reliable measure of the cognitive influences of intention it is acknowledged that utilising this theory in the current research does not take into account influences such as: potential physiological dependence [52]; or external influences such as a change of responsibility as women age that demands a changing of drinking pattern ([53]; e.g. older women adapt their consumption as a result of work and parenting demands, so that frequent drinking is more likely than binge drinking which can impact upon meeting responsibilities the next day). However, in line with the scope of the current study, the TPB is a fundamental and measurable model of an individual's intentions to drink as a function of their alcohol-related beliefs. Furthermore, although the TPB is a well-supported framework, future research may benefit from the investigation of additional normative influences such as those outlined in the Integrated Behavioral Model (IBM), an extension of the TPB [54]. The IBM defines normative beliefs as not just what an individual perceives other’s expectations to be, but also whether or not the individual perceives others to be engaging in the behavior as well. Previous research has incorporated both the TPB and IBM in an attempt to capture the potential variance of descriptive norms as defined by the IBM [55].

Overall, however, the current study has increased understanding of women’s decision-making around intentions to drink, specifically pertaining to three drinking behaviors. It has provided a picture of the underlying beliefs and patterns that significantly influence women of varying ages intentions to low risk drink, frequent drink and binge drink. Such an understanding can help target preventative measures for those women at risk of alcohol-related harms or interventions for those who are currently partaking in risky drinking practices.

## Conclusions

Given the existing knowledge that women’s drinking is emerging as a major issue across all age groups, this study has sought to identify how a woman’s intentions to drink and the underlying beliefs predictive of those intentions may change over their lifespan. The current study examined the underlying beliefs which predict women’s intentions to consume alcohol and engagement in drinking behavior. The current study provided evidence that the beliefs underlying intentions varied according to age group and drinking behavior. The presence of differing beliefs that underlie women’s motivations to drink according to age group and drinking behavior demonstrated the importance of tailoring interventions and public health messages to address specific age groups and drinking behaviors, thus targeting those most at risk and the most salient motivations underpinning their behavior. Additionally, identification of patterns across drinking behaviors and age has increased understanding of interventions that can provide a broader application. Drinking as a stress management tool was highlighted, as was women’s increased awareness of the short term effects of risky drinking behaviors, but seemingly limited awareness of the long term effects.

Overall, this study has addressed a number of gaps in the literature with regard to women’s drinking using a novel approach of integrating a well-known theoretical model to underpin a large sample size of empirical data (i.e. addressing the fluctuating drinking patterns with regard to different age cohorts; addressing intentions to consume alcohol in three different ways; and examining the significant underlying beliefs predictive of women’s intentions to consume alcohol). The key beliefs identified through a substantial cohort of women of various ages has been identified as important foci for future targeted interventions such as public health messages to address specific age groups and drinking behaviors, rather than a one size fits all approach.

## Abbreviation

TPB, The Theory of Planned Behavior

## Additional files

Additional file 1: Main Questionnaire. (PDF 584 kb) Additional file 2: Follow-up Questionnaire. (PDF 375 kb) Additional file 3: Pearson Correlation Matrices. (DOCX 50 kb)

## Footnotes

1 Checks were conducted to determine any potential differences between participants who completed either the online or paper version of the survey. No significant differences were found (e.g. education, Alcohol Use Disorders Identification Test between online and paper versions) and therefore responses from the two survey versions were combined.
