# Supplementary material for: Beliefs underlying Women’s intentions to consume alcohol
Source: BMC Womens Health. 2016 Jul 13;16:36. doi: 10.1186/s12905-016-0317-3 (PMC4943002; doi:10.1186/s12905-016-0317-3)
Supplement: Additional file 1: — Main Questionnaire. (PDF 584 kb) [file 12905_2016_317_MOESM1_ESM.pdf]

About the women's drinking behaviour project

We are conducting research looking at alcohol consumption by women 18 years of age **and above** who are living in Australia. Specifically, we are researching a range of alcohol consumption, not just problematic drinking. While males drink more frequently and in greater quantities than females, data from The Australian Bureau of Statistics, 2012, revealed that both younger and older women were starting to consume alcohol at risky or high-risk levels. While studies show that women's drinking (alcohol) is widespread and occurring at increasingly harmful levels, there is as yet little research examining the cultural and social influences on women's drinking. By exploring the place of alcohol in women's lives, and the factors that influence its consumption, the current research is a step in understanding women's drinking patterns.

What do we mean by 'Drinking'?

Drinking is the term we will use throughout this questionnaire to refer to consuming alcohol.
Drinking does NOT necessarily mean getting drunk, but will be used in one of three ways as defined below.

What do we mean by a Standard Drink?

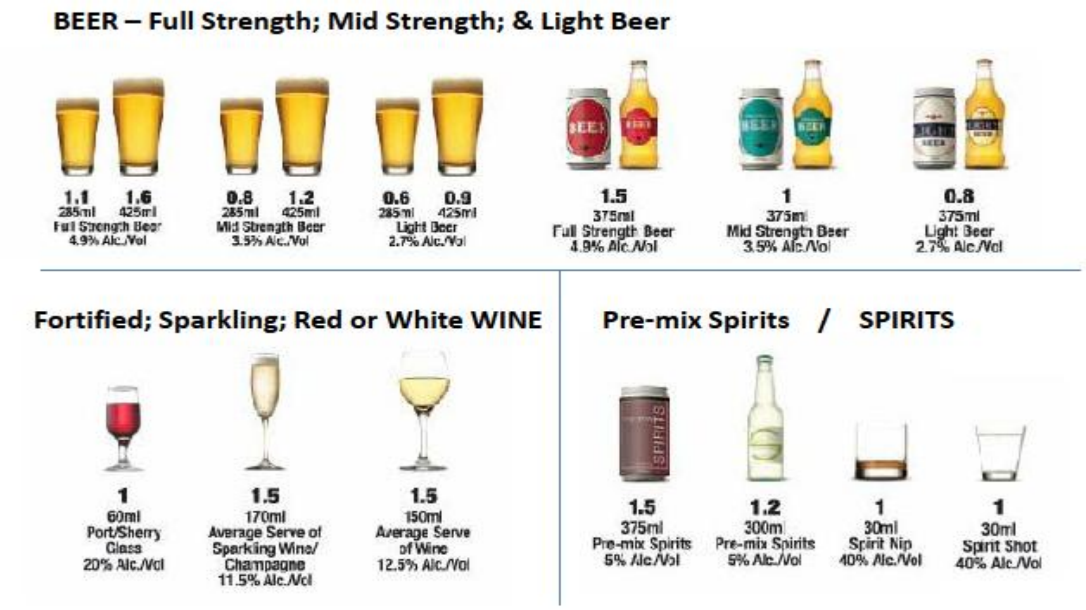

Adapted from Queensland Health Standard Drink Guide
http://www.health.qld.gov.au/cdg/docs/cdg_drink_guide.pdf

3 Definitions of drinking:

Two definitions of drinking will be used in this questionnaire:

1. Drinking in general, so this could mean occasional Drinking of one or two standard alcoholic beverages every few months or every week.
2. Drinking an alcoholic drink more than 5 days per week. (So for example, drinking one glass of wine per night, each night would be included in this definition.)
3. Drinking more than 4 standard drinks per drinking occasion. (So for example, drinking a bottle of wine or 3 large cocktails on a Friday or Saturday night.)

Your participation

Your participation involves completing 2 surveys (If you would prefer a paper version please email the principal researcher at hm.haydon@qut.edu.au). The first survey will take approximately 30 minutes to complete and is designed to assess your opinions about drinking alcohol. The second survey will be sent two weeks later and takes less than 5 minutes to complete. It would be greatly appreciated if you completed both surveys. For each of the survey questions please select the number that best represents your responses. The survey is about your beliefs and attitudes and therefore there is **no right or wrong answer.** Your answers are all **confidential**. We are interested in what you think about the following:

1. Your attitudes and beliefs about women's drinking in general and about your own drinking.
2. The things that might stop you from drinking alcohol.
3. The things that might make increase your drinking.
4. Your actual alcohol consumption.

Are you eligible to complete this survey?

To be eligible to complete this survey, you must meet the following criteria. Please tick the relevant boxes to confirm your eligibility.

1. I am female . ❑ Yes ❑ No
2. I am over 18 years old ❑ Yes ❑ No
3. I live in Australia ❑ Yes ❑ No

If you answered 'yes' to all questions, please proceed to the survey.

If you were not able to answer 'yes' to all three questions, you are not eligible to complete the survey. Thank you for your time.

Your Code Identifier

A code identifier helps to keep your identity confidential, but allows us to match two surveys together, so that the research team can match and analyse two surveys that are completed by the same person, but without knowing who completed the surveys. **Please put the appropriate letter or number in the left hand column below.**

	**What is the first letter of your first name?**
	**What is the third letter of your first name?**
	**What is the first letter in your mother's first name?**
	**What is the date of the month that you were born on? (e.g. the 21st)**

For example, imagine that Charlotte Brown is filling out the questionnaire. Her mother's name is Anne. Charlotte was born on the 31st of December. Her code identifier would be:

**C**	**A**	**A**	**31**

Section A: Information about you. (Remember all answers are strictly confidential. These questions are not intended to identify you in any way, but inform us of who is participating in the surveys.)

Sociodemographic information

Current Age: _______________ (years)

Which state/ territory do you currently live in? [Drop down Menu]

Is English your first language? ❑ Yes ❑ No

Do you identify as an Aboriginal and/or Torres Strait Islander woman? ❑ Yes ❑ No

Q1: What is your marital status?

❑ Never married
❑ Married/Defacto
❑ Divorced
❑ Widowed

Q2: What is your highest education level attained?

❑ Primary school
❑ High school/ Secondary school
❑ Technical/trade certificate/ Diploma
❑ University - undergraduate degree
❑ University - postgraduate degree

Section B: Information about your drinking. (Remember that all answers are strictly confidential.) *the AUDIT (Alcohol Use Disorders Identification Test)*

Q3. How often do you have a drink containing alcohol?
· Never
· Monthly or less
· 2-4 times a month
· 2-3 times a week
· 4 or more times a week

Q44. How many standard drinks containing alcohol do you have on a typical day when drinking?
· 1 or 2
· 3 or 4
· 5 or 6
· 7 to 9
· 10 or more

Q5. How often do you have six or more drinks on one occasion?
· Never
· Less than monthly
· Monthly
· Weekly
· Daily or almost daily

Q6. How often do you have five or more drinks on one occasion?
· Never
· Less than monthly
· Monthly
· Weekly
· Daily or almost daily

Q7. During the past year, how often have you found that you were not able to stop drinking once you had started?
· Never
· Less than monthly
· Monthly
· Weekly
· Daily or almost daily

Q8. During the past year, how often have you failed to do what was normally expected of you because of drinking?
· Never
· Less than monthly
· Monthly
· Weekly
· Daily or almost daily

Q9. During the past year, how often have you needed a drink in the morning to get yourself going after a heavy drinking session?

· Never
· Less than monthly
· Monthly
· Weekly
· Daily or almost daily

Q10. During the past year, how often have you had a feeling of guilt or remorse after drinking?

· Never
· Less than monthly
· Monthly
· Weekly
· Daily or almost daily

Q11. During the past year, have you been unable to remember what happened the night before because you had been drinking?

· Never
· Less than monthly
· Monthly
· Weekly
· Daily or almost daily

Q12. Have you or someone else been injured as a result of your drinking?

· No
· Yes, but not in the past year
· Yes, during the past year

Q13. Has a relative or friend, doctor or other health worker been concerned about your drinking or suggested you cut down?

· No
· Yes, but not in the past year
• Yes, during the past year

Section C: Your beliefs about drinking alcohol in general (Remember that all answers are strictly confidential.) *TPB questions*

Q14: How likely is it that the following people would think that you <u>should</u> drink on a regular basis? (Please circle the appropriate number on each row)

	extremely unlikely	quite unlikely	slightly unlikely	neither unlikely nor likely	slightly likely	quite likely	extremely likely	not applicable
My spouse/partner	1	2	3	4	5	6	7	8
My family	1	2	3	4	5	6	7	8
My friends	1	2	3	4	5	6	7	8
My professional/ work colleagues	1	2	3	4	5	6	7	8

Q15: How likely is it that the following would occur as a result of you drinking alcohol?
(Please circle the appropriate number on each row)

**Having a drink containing alcohol would ..**	extremely unlikely	quite unlikely	slightly unlikely	neither unlikely nor likely	slightly likely	quite likely	extremely likely
Help me to talk with others	1	2	3	4	5	6	7
Make me appear to be a fun person	1	2	3	4	5	6	7
Help me to have fun	1	2	3	4	5	6	7
Help me to socialise with others	1	2	3	4	5	6	7
Make me sick in the short term	1	2	3	4	5	6	7
Make me feel relaxed	1	2	3	4	5	6	7
Cause ill-health in the long term	1	2	3	4	5	6	7
Help me to unwind	1	2	3	4	5	6	7
Stop me from meeting people	1	2	3	4	5	6	7
Cost more than I can afford	1	2	3	4	5	6	7
Interfere with my existing commitments	1	2	3	4	5	6	7
Interfere with my family life	1	2	3	4	5	6	7
Make me feel irresponsible	1	2	3	4	5	6	7

Make me less boring	1	2	3	4	5	6	7
Help me to find a partner	1	2	3	4	5	6	7
Make me lose too much control	1	2	3	4	5	6	7

Q16: How likely is it that the following would STOP you from drinking alcohol?

(Please circle the appropriate number on each row)

**The following factors would stop me from drinking …**	extremely unlikely	quite unlikely	slightly unlikely	neither unlikely nor likely	slightly likely	quite likely	extremely likely
Having long term health issues	1	2	3	4	5	6	7
Having concerned family members or friends	1	2	3	4	5	6	7
Having family commitments that I must keep	1	2	3	4	5	6	7
Having other (non family) commitments that I must keep	1	2	3	4	5	6	7
Having to drive	1	2	3	4	5	6	7
Being pregnant or breastfeeding	1	2	3	4	5	6	7
Having short term side effects from alcohol (e.g. hangovers)	1	2	3	4	5	6	7
Not being able to control my behaviour when I drink	1	2	3	4	5	6	7
Being uncomfortable (e.g. uncomfortable feelings, environment)	1	2	3	4	5	6	7
Being at risk from others	1	2	3	4	5	6	7
Having beliefs or values (e.g. health, or sport motivation, religious or spiritual) that do not align with drinking	1	2	3	4	5	6	7
Being a mother	1	2	3	4	5	6	7
Having to travel too far to buy alcohol.	1	2	3	4	5	6	7

Q17: How likely is it that the following would INCREASE the possibility of you drinking alcohol? (Please circle the appropriate number on each row)

**The following would increase the chances of me drinking...**	extremely unlikely	quite unlikely	slightly unlikely	neither likely nor unlikely	somewhat likely	quite likely	extremely likely
If the drinking venues or restaurants were in walking distance.	1	2	3	4	5	6	7
If I have had a really stressful week.	1	2	3	4	5	6	7
If my tolerance for alcohol meant that I didn't "suffer" from ill effects the next day.	1	2	3	4	5	6	7
If it is Christmas time or New Year.	1	2	3	4	5	6	7
If alcohol was more affordable.	1	2	3	4	5	6	7
If I am on holidays.	1	2	3	4	5	6	7
If I didn't have to get up so early in the mornings.	1	2	3	4	5	6	7
If I was single and looking for a partner.	1	2	3	4	5	6	7
If it was Melbourne Cup Day or another important sporting event.	1	2	3	4	5	6	7
If I didn't have children.	1	2	3	4	5	6	7
Going to a nice restaurant	1	2	3	4	5	6	7
If I was close to wineries or wine clubs	1	2	3	4	5	6	7
If it was my birthday.	1	2	3	4	5	6	7

This section is about drinking when it is drinking one or two drinks occasionally. So that may be if you have a glass of wine or two every week, or it may be that you have a champagne only on special occasions.

Q18: Thinking about drinking 1 or 2 drinks occasionally...

Do you agree that in the next 2 weeks…	Strongly disagree	disagree	Somewhat disagree	neither agree or disagree	slightly agree	agree	Strongly agree
It is likely that I will engage in drinking 1 or 2 drinks occasionally.	1	2	3	4	5	6	7
I intend to drink 1 or 2 drinks occasionally.	1	2	3	4	5	6	7
I plan to drink 1 or 2 drinks occasionally.	1	2	3	4	5	6	7
I never intend to drink 1 or 2 drinks occasionally, but I usually end up doing so	1	2	3	4	5	6	7

This section is about drinking 6 or more days in the one week. So that may be if you have a glass of wine or a beer most nights in a particular week. It may be that some weeks you have hardly any, but some weeks you have alcohol almost every day.

Q19: Thinking about drinking 6 or more days per week...

Do you agree that in the next 2 weeks…	Strongly disagree	disagree	Somewhat disagree	neither agree or disagree	slightly agree	agree	Strongly agree
It is likely that I will engage in drinking on 6 or more days in a typical week.	1	2	3	4	5	6	7
I intend to drink more than 5 days in a typical week.	1	2	3	4	5	6	7
I plan to drink more than 5 days in a typical week.	1	2	3	4	5	6	7
I never intend to drink more than 5 days in a week, but somehow I end up doing so	1	2	3	4	5	6	7

This section is about drinking 5 or more standard alcoholic drinks on any one occasion (or more than 4 drinks). For instance it could be when you drink 5 glasses of champagne at a barbecue or dinner party; or perhaps 5 cocktails on a Friday night.

Q20: Thinking about drinking more than 4 standard drinks on one occasion

Do you agree that in the next 2 weeks…	Strongly disagree	disagree	Somewhat disagree	neither agree or disagree	slightly agree	agree	Strongly agree
It is likely that I will drink more than 4 drinks on any one occasion	1	2	3	4	5	6	7
I intend to drink more than 4 drinks on one occasion	1	2	3	4	5	6	7
I plan to drink more than 4 drinks on one occasion.	1	2	3	4	5	6	7
On the weekend I plan to drink as much as I can without spending too much money	1	2	3	4	5	6	7
I never intend to drink more than 4 drinks in an evening, but somehow I end up doing so	1	2	3	4	5	6	7
