# Supplementary material for: Beliefs underlying Women’s intentions to consume alcohol
Source: BMC Womens Health. 2016 Jul 13;16:36. doi: 10.1186/s12905-016-0317-3 (PMC4943002; doi:10.1186/s12905-016-0317-3)
Supplement: Additional file 2: — Follow-up Questionnaire. (PDF 375 kb) [file 12905_2016_317_MOESM2_ESM.pdf]

About this women’s drinking behaviour project

We are conducting research looking at alcohol consumption by women 18 years of age **and above** living in Australia. Specifically, we are researching a range of alcohol consumption, not just problematic drinking. While males drink more frequently and in greater quantities than females, data from The Australian Bureau of Statistics, 2012, revealed that both younger and older women were starting to consume alcohol at risky or high-risk levels. While studies show that women’s drinking (alcohol) is widespread and occurring at increasingly harmful levels, there is as yet little research examining the cultural and social influences on women’s drinking. By exploring the place of alcohol in women's lives, and the factors that influence its consumption, the current research is a step in understanding women's drinking patterns.

What do we mean by a Standard Drink?

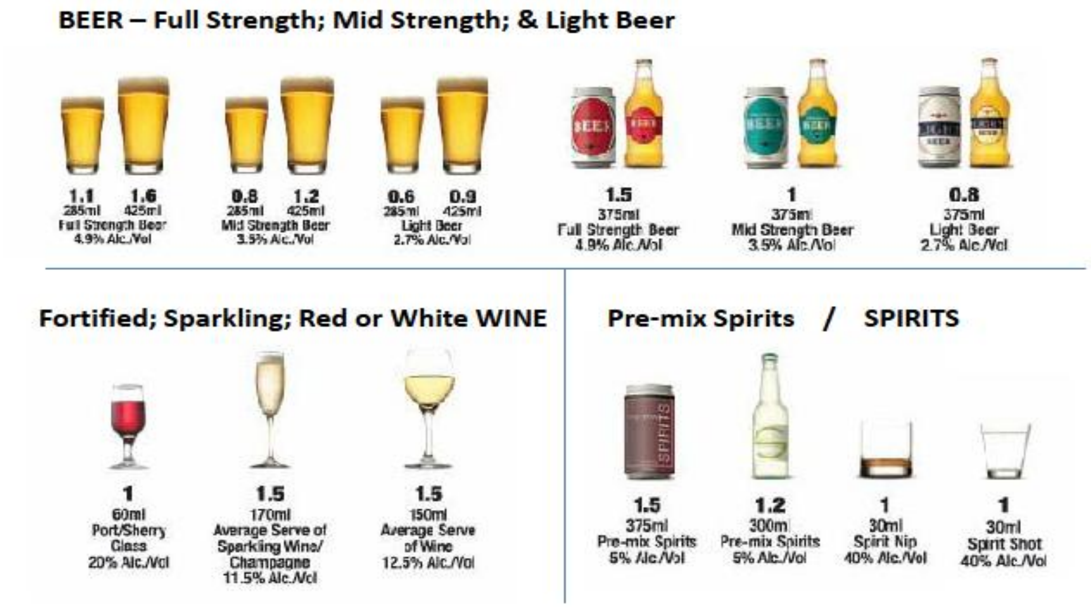

Adapted from Queensland Health Standard Drink Guide
http://www.health.qld.gov.au/cdg/docs/cdg_drink_guide.pdf

Your participation

This is the second and final survey that has 3 questions about your ACTUAL drinking behaviour in the past 2 weeks. This survey will take less than 5 minutes to complete. It would be greatly appreciated if you completed this final survey. There is **no right or wrong answer** and your answers are all **confidential**.

Are you eligible to complete this survey?

To be eligible to complete this survey, you must have completed the first survey approximately two weeks ago.

Your Code Identifier

So that we can match up this survey's responses with the previous responses please complete the code identifier as you did on Survey 1. Please put the appropriate letter or number in the left hand column below..

	**What is the first letter of your first name?**
	**What is the third letter of your first name?**
	**What is the first letter in your mother's first name?**
	**What is the date of the month that you were born on? (e.g. the 21st)**

For example, imagine that Charlotte Brown is filling out the questionnaire. Her mother's name is Anne. Charlotte was born on the 31st of December. Her code identifier would be:

**C**	**A**	**A**	**31**

Section A: Information about you. (Remember all answers are strictly confidential.)

Current Age: _______________ (years)

Country of birth: ____

Q1: In the last 2 weeks, on how many days did you have a drink containing alcohol?

0/None	1	2	3	4	5	6	7	8	9	10	11	12	13	14/ Every Day

Q2: In the last 2 weeks if you drank alcohol, how many standard alcoholic drinks did you have on a typical drinking occasion?

0/None	1	2	3	4	5	6	7	8	9	10 or More

Q3: In the last 2 weeks how often did you have <u>5 or more</u> standards drinks on any one occasion?

Never - 0	Once - 1	Twice - 2	3 times	4 times	5 or more times

Q4: In the last 2 weeks how often did you have <u>6 or more</u> standards drinks on any one occasion?

Never - 0	Once - 1	Twice - 2	3 times	4 times	5 or more times

Thank you very much for completing this survey.
