# Supplementary material for: Beliefs underlying Women’s intentions to consume alcohol
Source: BMC Womens Health. 2016 Jul 13;16:36. doi: 10.1186/s12905-016-0317-3 (PMC4943002; doi:10.1186/s12905-016-0317-3)
Supplement: Additional file 3: — Pearson Correlation Matrices. (DOCX 50 kb) [file 12905_2016_317_MOESM3_ESM.docx]

Appendices

Appendix A

Table A1

*Correlations of Beliefs with Intentions to Low Risk Drink across Age Groups*

|  | Pearsons correlation (r) for each age group | | | | |
| --- | --- | --- | --- | --- | --- |
| Beliefs | 18 to 24 | 25 to 34 | 35 to 44 | 45 to 54 | 55 Plus |
| Behavioral beliefs  Benefits  Make me appear to be a fun person  Help me to have fun  Help me to socialise with others  Make me feel relaxed  Help me to unwind  Make me less boring  Help me to find a partner  Help me to talk with others  Costs  Make me sick in the short term  Cause ill-health in the long term  Stop me from meeting people  Cost more than I can afford  Interfere with my existing commitments  Interfere with my family life  Make me feel irresponsible  Make me lose control  Normative beliefs  Spouse/ partner  Family  Friends  Professional/ Work colleagues  Having concerned family members or friends  Control beliefs  Barriers  Having health issues  Having family commitments that I must keep  Having non family commitments I must keep  Having to drive  Being pregnant or breastfeeding  Short term side effects of alcohol (hangovers)  Not able to control my behavior when I drink  Being uncomfortable (e.g. feelings, in an environment)  Being at risk from others  Beliefs or values (e.g. health, sport or religious) not aligned with drinking  Being a mother  Having to travel too far to buy alcohol.  Facilitators  If drinking venues were in walking distance  If I have had a really stressful week.  If my tolerance for alcohol meant that I didn’t “suffer” from ill effects the next day  If it is Christmas /New Year (Cultural Celebration).  If alcohol was more affordable  If I am on holidays  If I didn’t have to get up so early in the mornings  If I was single and looking for a partner  If it was Melbourne Cup Day or another important sporting event  If I didn’t have children  If I was at a nice restaurant  If I was close to wineries or wine clubs  If it was my birthday. | .320**  .374**  .343**  .383**  .419**  .246**  .213**  .253**  -.010  -.130*  .076  -.049  -.179**  -.134*  -.069  -.070  .125*  .150**  .126*  .074  -.182**  -.104  -.091  -.106  .013  .071  -.276**  -.249**  -.255**  -.150**  -.243**  -.092  .030  .371**  .461**  .451**  .563**  .483**  .638**  .430**  .377**  .365**  .392**  .461**  .319**  .591** | .091  .270**  .256**  .320**  .322**  .151*  -.006  .106  -.142*  -.170**  -.083  -.158**  -.215**  -.299**  -.179**  -.149*  .154*  -.001  .196**  .069  -.048  .016  -.083  -.100  -.015  .080  -.224**  -.120*  -.005  .005  -.076  -.100  .013  .326**  .362**  .177**  .400**  .213**  .366**  .206**  .197**  .334**  .224**  .421**  .209**  .449** | .153*  .256**  .209**  .426**  .378**  .162*  .046  .174*  -.215**  -.214**  -.025  -.213**  -.305**  -.333**  -.159*  -.184**  .144*  .007  .113  -.036  -.033  -.099  .074  .120  .206**  .027  -.092  .079  -.006  .105  -.077  -.186**  -.006  .257**  .403**  .272**  .426**  .191**  .512**  .204**  .252**  .237**  .212**  .395**  .225**  .436** | .033  .156  .213**  .437**  .379**  -.004  -.139  .067  -.348**  -.226**  -.039  -.110  -.127  -.166*  -.114  -.190*  -.033  -.072  -.074  -.102  .073  .154  .068  .089  .193*  .088  .036  .209*  .059  .146  .010  -.224**  .052  .019  .364**  .116  .421**  .027  .415**  .145  .029  .125  .086  .422**  .166*  .410** | .097  .214*  .164  .192  .208*  .042  .147  .190  -.267**  -.199  .061  -.134  -.257*  -.316**  -.190  -.174  .246/*  .152  .145  .001  -.107  .048  .093  .134  .049  -.009  -.114  -.051  .021  .036  .002  -.088  .138  .324**  .370**  .383**  .403**  .353**  .385**  .236*  .170  .331**  .300**  .385**  .475**  .391** |

** Correlation is significant at the 0.01 level (2-tailed). * Correlation is significant at the 0.05 level (2-tailed).

Table A2

*Correlations of Beliefs with Intentions to Frequent Drink across Age Groups*

|  | Pearsons correlation (r) for each age group | | | | |
| --- | --- | --- | --- | --- | --- |
| Beliefs | 18 to 24 | 25 to 34 | 35 to 44 | 45 to 54 | 55 Plus |
| Behavioral beliefs  Benefits  Make me appear to be a fun person  Help me to have fun  Help me to socialise with others  Make me feel relaxed  Help me to unwind  Make me less boring  Help me to find a partner  Help me to talk with others  Costs  Make me sick in the short term  Cause ill-health in the long term  Stop me from meeting people  Cost more than I can afford  Interfere with my existing commitments  Interfere with my family life  Make me feel irresponsible  Make me lose control  Normative beliefs  Spouse/ partner  Family  Friends  Professional/ Work colleagues  Having concerned family members or friends  Control beliefs  Barriers  Having health issues  Having family commitments that I must keep  Having non family commitments I must keep  Having to drive  Being pregnant or breastfeeding  Short term side effects of alcohol (hangovers)  Not able to control my behavior when I drink  Being uncomfortable (e.g. feelings, in an environment)  Being at risk from others  Beliefs or values (e.g. health, sport or religious) not aligned with drinking  Being a mother  Having to travel too far to buy alcohol.  Facilitators  If drinking venues were in walking distance  If I have had a really stressful week.  If my tolerance for alcohol meant that I didn’t “suffer” from ill effects the next day  If it is Christmas /New Year (Cultural Celebration).  If alcohol was more affordable  If I am on holidays  If I didn’t have to get up so early in the mornings  If I was single and looking for a partner  If it was Melbourne Cup Day or another important sporting event  If I didn’t have children  If I was at a nice restaurant  If I was close to wineries or wine clubs  If it was my birthday. | .017  -.010  .000  .007  .067  -.004  .094  .022  -.037  .040  .128*  .165**  .084  .075  .030  -.019  .020  .114*  -.033  .101  -.181**  -.200**  -.217**  -.139*  -.245**  -.174**  -.039  -.092  -.123*  -.051  -.127*  -.070  -.050  .144**  .206**  .119*  .130*  .148**  .109  .113*  .091  .115*  .112*  .185**  .201**  .060 | .010  .146*  .118*  .170**  .244**  .012  .047  .118  -.115  .010  .036  -.027  .018  .098  .002  .026  .110  .021  .055  .006  -.319**  -.165**  -.107  -.169**  -.091  -.200**  -.115  -.110  -.246**  -.157**  -.106  -.194**  -.074  .083  .163**  .091  .080  .150*  .015  .196**  .052  .081  .133*  .048  .120*  .125* | .089  .173*  .116  .228**  .181**  .099  .115  .109  -.001  .108  .046  .023  .071  .068  -.031  .059  .033  .118  .041  .004  -.184**  -.131  -.102  -.167*  .007  -.208**  -.182**  -.072  -.051  -.002  -.080  -.073  .028  .159*  .235**  .218**  .163*  .216**  .174*  .194**  .109  .197**  .106  .177*  .178**  .166* | -.079  -.020  .052  .253**  .224**  -.026  .067  .048  -.132  .077  .061  -.089  -.073  .002  -.118  -.116  .113  -.001  .008  .069  -.263**  -.271**  -.230**  -.228**  .019  .001  -.107  -.001  -.027  .015  -.160  -.097  -.027  .066  .267**  .066  .013  .165*  .040  .074  .087  .042  .153  .050  .059  .009 | .138  .047  .064  .085  .068  .107  -.027  .047  -.040  .105  .053  .085  -.055  .009  .075  -.031  .069  .176  .216*  -.012  -.193  -.126  -.299**  -.249*  -.078  -.049  -.072  .029  -.127  -.019  .006  -.048  -.010  .065  .269**  .159  .157  .142  .092  .141  .023  .264**  .254*  .149  -.086  .151 |

** Correlation is significant at the 0.01 level (2-tailed). * Correlation is significant at the 0.05 level (2-tailed).

Table A3

*Correlations of Beliefs with Intentions to Binge Drink across Age Groups*

|  | Pearsons correlation (r) for each age group | | | | |
| --- | --- | --- | --- | --- | --- |
| Beliefs | 18 to 24 | 25 to 34 | 35 to 44 | 45 to 54 | 55 Plus |
| Behavioral beliefs  Benefits  Make me appear to be a fun person  Help me to have fun  Help me to socialise with others  Make me feel relaxed  Help me to unwind  Make me less boring  Help me to find a partner  Help me to talk with others  Costs  Make me sick in the short term  Cause ill-health in the long term  Stop me from meeting people  Cost more than I can afford  Interfere with my existing commitments  Interfere with my family life  Make me feel irresponsible  Make me lose control  Normative beliefs  Spouse/ partner  Family  Friends  Professional/ Work colleagues  Having concerned family members or friends  Control beliefs  Barriers  Having health issues  Having family commitments that I must keep  Having non family commitments I must keep  Having to drive  Being pregnant or breastfeeding  Short term side effects of alcohol (hangovers)  Not able to control my behavior when I drink  Being uncomfortable (e.g. feelings, in an environment)  Being at risk from others  Beliefs or values (e.g. health, sport or religious) not aligned with drinking  Being a mother  Having to travel too far to buy alcohol.  Facilitators  If drinking venues were in walking distance  If I have had a really stressful week.  If my tolerance for alcohol meant that I didn’t “suffer” from ill effects the next day  If it is Christmas time or New Year (Cultural Celebration).  If alcohol was more affordable  If I am on holidays  If I didn’t have to get up so early in the mornings  If I was single and looking for a partner  If it was Melbourne Cup Day or another important sporting event  If I didn’t have children  If I was at a nice restaurant  If I was close to wineries or wine clubs  If it was my birthday. | .372**  .441**  .408**  .379**  .416**  .321**  .275**  .326**  .084  -.106  -.028  -.047  -.065  -.129*  -.031  .142*  .115*  .125*  .255**  .061  -.243**  -.211**  -.175**  -.209**  -.080  .021  -.524**  -.421**  -.443**  -.288**  -.250**  -.061  -.016  .419**  .434**  .536**  .481**  .522**  .486**  .488**  .442**  .403**  .523**  .257**  .206**  .566** | .385**  .430**  .338**  .270**  .325**  .373**  .303**  .272**  .097  .110  .108  .090  .141*  -.012  .098  .183**  .305**  .109  .254**  .083  -.240**  -.119*  -.147*  -.212**  -.004  .130*  -.416**  -.256**  -.226**  -.096  -.226**  .008  -.015  .361**  .289**  .362**  .288**  .349**  .318**  .353**  .335**  .421**  .260**  .191**  .205**  .405** | .195**  .336**  .289**  .315**  .339**  .202**  .262**  .172*  .032  .015  -.002  -.009  -.043  -.080  .032  .114  .194**  .072  .179**  .046  -.187**  -.222**  .010  -.023  .031  .028  -.303**  -.178**  -.147*  -.052  -.184**  -.175*  -.121  .347**  .304**  .285**  .359**  .313**  .423**  .315**  .301**  .450**  .196**  .235**  .203**  .392** | .018  .254**  .219**  .365**  .303**  .132  .183*  .258**  -.002  .037  .171*  -.110  -.047  -.001  .030  .039  .138  .026  .061  .003  -.328**  -.239**  -.227**  -.230**  -.221**  -.262**  -.412**  -.248**  -.330**  -.264**  -.389**  -.231**  -.179*  .268**  .282**  .215**  .165*  .167*  .260**  .228**  .141  .142  .165*  .086  .100  .183* | .260**  .244*  .226*  .073  .174  .109  .103  .133  .036  .178  .180  .064  .066  .203*  .258*  .241*  -.023  .111  .092  .008  -.100  -.117  -.145  -.246*  -.076  -.154  -.252*  -.169  -.200  -.103  -.028  .024  -.013  -.095  .232*  .229*  .124  .099  .055  .107  .132  .225*  .152  .059  -.073  .148 |

*** *p* <.001 ** *p* <.01 * *p* <.05

Appendix B

Table B1

*Summary of Stepwise Regression results Identifying Critical-Belief based Predictors of Intention to Low Risk Drink.*

|  | β | | | | |
| --- | --- | --- | --- | --- | --- |
| Beliefs predicting intentions to low risk drink | 18 to 24 | 25 to 34 | 35 to 44 | 45 to 54 | 55 Plus |
| Behavioral beliefs  Benefits  Help me to have fun  Make me feel relaxed  Help me to unwind  Costs  Make me sick in the short term  Interfere with my existing commitments  Interfere with my family life  Normative beliefs  Spouse/ partner  Family  Friends  Control beliefs  Barriers  Having to drive  Being pregnant or breastfeeding  Short term side effects of alcohol (hangovers)  Not able to control my behavior when I drink  Being uncomfortable (e.g. feelings, in an environment)  Beliefs or values (e.g. health, sport or religious) not aligned with drinking  Being a mother  Facilitators  If I have had a really stressful week.  If it is Christmas time or New Year (Cultural Celebration).  If I am on holidays  If I was at a nice restaurant  If I was close to wineries or wine clubs  If it was my birthday. | .20**  .31***  -.18**  .15**  -.18**  -.14*  -.13*  .37***  .15**  .26*** | .33***  -.30***  .20**  -.22***  .14*  .24***  .26*** | .43***  -.34**  .14*  .23**  -.22**  .17*  .42*** | .44***  -.36***  .30***  -.31***  .22**  .35*** | .21*  -.32**  .25*  .25**  .40*** |

*** *p* <.001 ** *p* <.01 * *p* <.05

Table B2

*Summary of Stepwise Regression results Identifying Critical-Belief based Predictors of Intention to Frequent Drink.*

|  | β | | | | |
| --- | --- | --- | --- | --- | --- |
| Beliefs predicting intentions to frequent drink | 18 to 24 | 25 to 34 | 35 to 44 | 45 to 54 | 55 Plus |
| Behavioral beliefs  Benefits  Make me feel relaxed  Help me to unwind  Costs  Cost more than I can afford  Normative beliefs  Family  Friends  Having concerned family members or friends  Control beliefs  Barriers  Having health issues  Having family commitments that I must keep  Having to drive  Being pregnant or breastfeeding  Short term side effects of alcohol (hangovers)  Being uncomfortable (e.g. feelings, in an environment)  Facilitators  If I have had a really stressful week.  If alcohol was more affordable  If I didn’t have to get up so early in the mornings  If I was close to wineries or wine clubs | .17**  .11*  -.13*  -.20***  .13*  .14* | .24***  -.22**  -.13*  -.13*  .20** | .23**  -.19**  -.16*  .16*  .15* | .25**  -.32***  .27** | .22*  -.32*  .27** |

*** *p* <.001 ** *p* <.01 * *p* <.05

Table B3

*Summary of Stepwise Regression results Identifying Critical-Belief based Predictors of Intention to Binge Drink.*

|  | β | | | | |
| --- | --- | --- | --- | --- | --- |
| Beliefs predicting intentions to binge drink | 18 to 24 | 25 to 34 | 35 to 44 | 45 to 54 | 55 Plus |
| Behavioral beliefs  Benefits  Make me appear to be a fun person  Help me to have fun  Help me to socialise with others  Make me feel relaxed  Help me to unwind  Help me to find a partner  Costs  Stop me from meeting people  Interfere with my family life  Make me feel irresponsible  Make me lose control  Normative beliefs  Spouse/ partner  Friends  Having concerned family members or friends  Control beliefs  Barriers  Having health issues  Having non family commitments I must keep  Being pregnant or breastfeeding  Short term side effects of alcohol (hangovers)  Being uncomfortable (e.g. feelings, in an environment)  Beliefs or values (e.g. health, sport or religious) not aligned with drinking  Having to travel too far to buy alcohol.  Facilitators  If drinking venues were in walking distance  If I have had a really stressful week.  If my tolerance for alcohol meant that I didn’t “suffer” from ill effects the next day  If alcohol was more affordable  If I am on holidays  If I was single and looking for a partner  If it was Melbourne Cup Day or another important sporting event  If I didn’t have children  If I was close to wineries or wine clubs  If it was my birthday. | .21**  .16*  .22**  -.18**  .19**  .26***  -.42***  -.29***  .18**  .18**  .12*  .21***  -.16**  .26*** | .36***  .20**  .18**  .24***  .16*  -.19**  .26***  -.387***  .17**  .15*  .24***  .17** | .21**  .14*  .19**  -.15*  -.26***  .26***  .32*** | .36***  .17*  .17*  -.17*  -.24**  -.25**  .18*  .21* | .26*  .26*  -.25*  .23* |

*** *p* <.001 ** *p* <.01 * *p* <.05
